# Dr. M. S. Dean

**Published:** 1882-02

**Authors:** 


*Death of Dr. M. S. Dean.*—The sad news of the loss of one of the most eminent members of the dental profession, which occurred suddently in Chicago a few days ago reached us too late for an extended notice in the present number of the Journal. The information is derived from a daily paper and next month we will give a more extended notice.

